# Crystal structures of the complexes containing macrocyclic cations [*M*(cyclam)]^2+^ (*M* = Ni, Zn) and tetra­iodido­cadmate(2–) anion

**DOI:** 10.1107/S2056989023007004

**Published:** 2023-08-23

**Authors:** Irina L. Andriichuk, Sergiu Shova, Yaroslaw D. Lampeka

**Affiliations:** a L. V. Pisarzhevskii Institute of Physical Chemistry of the National Academy of Sciences of Ukraine, Prospekt Nauki 31, 03028, Kiev, Ukraine; b "Petru Poni" Institute of Macromolecular Chemistry, Department of Inorganic Polymers, Aleea Grigore Ghika Voda 41A, RO-700487 Iasi, Romania; Universidade de Sâo Paulo, Brazil

**Keywords:** crystal structure, nickel, zinc, cyclam, tetra­iodo­cadmate

## Abstract

The asymmetric units of the isostructural compounds (1,4,8,11-tetra­aza­cyclo­tetra­decane-κ^4^ *N*)nickel(II) tetra­iodido­cadmate(II), [Ni(C_10_H_24_N_4_)][CdI_4_] (**I**), and tri­iodido-1κ^3^ *I*-μ-iodido-(1,4,8,11-tetra­aza­cyclo­tetra­decane-2κ^4^ *N*)cad­mium(II)zinc(II), [CdZnI_4_(C_10_H_24_N_4_)] (**II**) (C_10_H_24_N_4_ = 1,4,8,11-tetra­aza­cyclo­tetra­decane, cyclam, *L*), consist of the centrosymmetric macrocyclic cation [*M*(*L*)]^2+^ [*M* = Ni^II^ or Zn^II^] with the metal ion lying on a twofold screw axis, and the tetra­iodo­cadmate anion [CdI_4_]^2−^ located on the mirror plane. In **I**, the anion acts as an uncoordinated counter-ion while in **II** it is bound to the Zn^II^ atom *via* one of the iodide atoms, thus forming an electroneutral heterobimetallic complex [Zn(*L*)(CdI_4_)]. The Ni^II^ and Zn^II^ ions are coordinated in a square-planar manner by the four secondary N atoms of the macrocyclic ligand *L*, which adopts the most energetically stable *trans*-III conformation. The [CdI_4_]^2−^ anions in **I** and **II** are structurally very similar and represent slightly deformed tetra­hedrons with average Cd—I bond lengths and I—Cd—I angles of *ca* 2.79 Å and 109.6°, respectively. The supra­molecular organization of the complexes under consideration in the crystals is very similar and is determined by the hydrogen-bonding inter­actions between the secondary amino groups of the ligand *L* in the [*M*(*L*)]^2+^ cations and iodide atoms of the [CdI_4_]^2−^ anion. In particular, the alternating cations and anions form chains running along the *b-*axis direction that are arranged into di-periodic sheets oriented parallel to the (101) and (  01) planes. Because both kinds of sheets are built from the same cations and anions, this feature provides the three-dimensional coherence of the crystals of **I** and **II**.

## Abstract 2

The isostructural compounds **I** and **II** are composed of planar macrocyclic cations [*M*(cyclam)]^2+^ and the tetra­hedral anion [CdI_4_]^2−^, which plays a purely charge-compensation function in the Ni^II^ complex **I** and is axially coordinated *via* the iodide atom in the Zn^II^ complex **II**. In both complexes, as a result of N–H⋯I hydrogen bonding, the alternating cations and anions form chains running along the *b*-axis direction that are arranged into di-periodic sheets oriented parallel to the (101) and ( 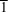 01) planes.

## 1. Chemical context

Iodo­cadmates are one of the representatives of organic–inorganic hybrid perovskites that have been studied intensively recently. They are characterized by a number of specific electric and optical properties (Rok *et al.*, 2021 ▸) that are dependent on the structure of the complex anions [Cd_*m*_I_*n*_]^(*n*−2*m*)−^ which, in turn, is determined by the structure of the organic or metallocomplex cation that is used as a structure-directing agent during the synthesis. Depending on this agent, in addition to the most common mononuclear [CdI_4_]^2–^ anion, several types of oligonuclear {[Cd_2_I_6_]^2–^ (Park *et al.*, 2018 ▸), [Cd_3_I_7_]^−^ (Bao *et al.*, 2013 ▸), [Cd_4_I_10_]^2–^ (Park *et al.*, 2014 ▸), [Cd_4_I_12_]^4–^ (Lee *et al.*, 2016 ▸), [Cd_6_I_16_]^4–^ (Bach *et al.*, 1997 ▸)} and polymeric (Dobrzycki & Wózniak, 2009 ▸; Sharutin *et al.*, 2012 ▸; Rok *et al.*, 2021 ▸) iodo­cadmates have been structurally characterized. In some cases, octa­hedral complexes of penta- and hexa­dentate macrocyclic ligands have been used as the structure-directing agents in Cd^II^–iodide systems (Lee *et al.*, 2016 ▸; Park *et al.*, 2018 ▸). At the same time, square-planar cations formed by the tetra­aza­macrocyclic ligand cyclam (cyclam = 1,4,8,11-tetra­aza­cyclo­tetra­decane, C_10_H_24_N_4_, *L*), which is the most suitable for binding of 3*d* transition-metal ions (Yatsimirskii & Lampeka, 1985 ▸) were never exploited in this respect, though the fruitfulness of such an approach was shown formerly during the preparation of iodo­plumbate hybrids containing the [Ni(TMC)]^2+^ cation (TMC = 1,4,8,11-tetra­methyl-1,4,8,11-tetra­aza­cyclo­tetra­deca­ne) (Zhang *et al.*, 2019 ▸). 
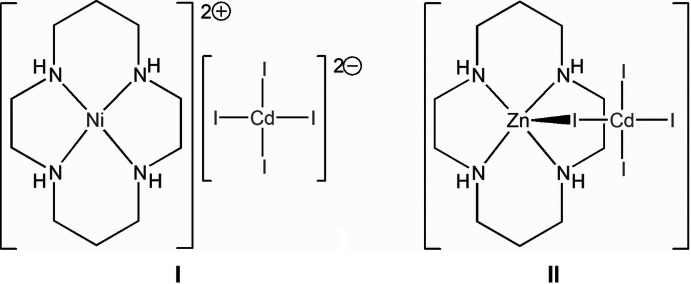


The present work describes the preparation and structural characterization of two representatives of iodo­cadmate hybrids formed under the structure-directing influence of the Ni^II^ and Zn^II^ cyclam complexes, namely (1,4,8,11-tetra­aza­cyclo­tetra­decane-κ^4^ *N*)nickel(II) tetra­iodido­cadmate(II), [Ni(C_10_H_24_N_4_)][CdI_4_] (**I**), and tri­iodido-1κ^3^ *I*-μ-iodido-(1,4,8,11-tetra­aza­cyclo­tetra­decane-2κ^4^ *N*)cadmium(II)zinc(II), [CdZnI_4_(C_10_H_24_N_4_)] (**II**).

## 2. Structural commentary

**Figure 1:**
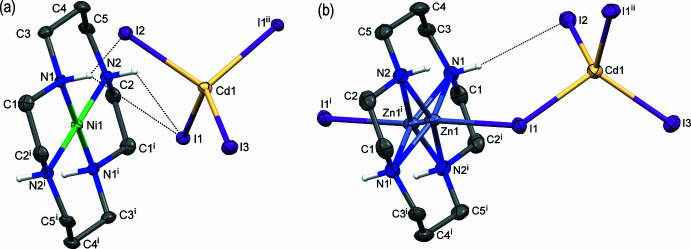
View of the mol­ecular structures of **I** and **II** showing the atom-labeling scheme, with displacement ellipsoids drawn at the 30% probability level. C-bound H atoms are omitted for clarity. Hydrogen-bonding inter­actions are shown as dotted lines. Symmetry codes: (i) −*x* + 1, −*y* + 1, −*z* + 1; (ii) *x*, −*y* + 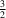 , *z*.

The asymmetric units of the isostructural compounds **I** and **II** involve the centrosymmetric macrocyclic cation [*M*(*L*)]^2+^ [*M* = Ni^II^ and Zn^II^, respectively] with the metal ions lying on a twofold screw axis and the tetra­iodo­cadmate anion [CdI_4_]^2−^. The latter acts as an uncoordinated counter-ion in **I** but is coordinated to the Zn^II^ in **II**, thus forming an electroneutral heterobimetallic complex [Zn(*L*)(CdI_4_)] in which the I1 atom plays a μ_2_-bridging function (Fig. 1 ▸). The Cd1, I2 and I3 atoms of the tetra­iodo­cadmate anions in **I** and **II** are located on the mirror plane. The [CdI_4_]^2−^ moieties as a whole represent slightly deformed tetra­hedrons with Cd—I bond lengths and I—Cd—I angles varying in the narrow ranges not exceeding 0.08 Å and 8.2°, respectively (Table 1 ▸).

The Ni^II^ ion in **I** is coordinated by the four secondary N atoms of the macrocycle *L* (Fig. 1 ▸ *a*) and the centrosymmetry of the cation ensures the strict planarity of the Ni(N_4_) coord­ination environment. The Ni—N bond lengths of *ca* 1.94 Å (Table 1 ▸) are typical of four-coordinated low-spin square-planar *d* ^8^ Ni^II^ complexes with macrocyclic 14-mem­bered tetra­amine ligands and are much shorter than those (*ca* 2.05 Å) observed in the high-spin six-coordinated tetra­gonal–bipyramidal macrocyclic species (Yatsimirskii & Lampeka, 1985 ▸). The macrocyclic ligand *L* in the complex cations of **I** adopts the most common and energetically favorable *trans*-III (*R*,*R*,*S*,*S*) conformation (Bosnich *et al.*, 1965*a* ▸; Barefield *et al.*, 1986 ▸). Its five- and six-membered chelate rings are present in *gauche* and *chair* conformations with the bite angles of *ca* 87 and 93°, respectively (Table 1 ▸).

The bifurcating hydrogen-bonding inter­action between the I1 atom of the anion and the secondary amino groups of the macrocyclic ligand of the cation as well as the N1—H1⋯I2 contact (Fig. 1 ▸ *a*, for parameters of the hydrogen bonds see Table 2 ▸) in **I** arrange the [CdI_4_]^2−^ fragment in such a way that its I1 atom is located just above the Ni(N_4_) plane in a potential axial position of the coordination sphere of the Ni^II^ ion (the deviation of the mean angles N—Ni1—I1 from 90° do not exceed 4°). However, the very long distance between the metal ion and this iodide [3.3618 (3) Å] allows a coordinative inter­action between them to be excluded. This is in agreement with the Ni—N bond lengths typical of the square-planar Ni^II^ species (see *Database survey*).

The mol­ecular structure of **II** is shown in Fig. 1 ▸ *b*. Similarly to the Ni^II^ atom in **I**, the Zn^II^ ion in the macrocyclic cation is coordinated by the four secondary N atoms of the macrocycle *L* but is displaced by 0.336 (1) Å from the N_4_ plane towards the apically coordinated I1 atom. Because the [Zn(*L*)] unit is centrosymmetric, the metal ion was found to be disordered around a center of inversion and thus was refined with half occupancy.

The weak coordination of the iodide atom in the axial position of the macrocyclic cation (Zn1—I1 bond length *ca* 2.9 Å, Table 1 ▸) is reinforced by the hydrogen-bonding inter­action N1—H1⋯I2 (Table 3 ▸) and results in the deformed square-pyramidal coordination environment of the Zn^II^ ion. Though the Zn—I—Cd angle [119.79 (4)°] and the mean Ni⋯I—Cd angle [120.13 (2)°] are practically identical, the displacement of the Zn^II^ ion from the mean N4 plane of the macrocycle and a shorter distance between Zn^II^ and the apical iodide than for Ni^II^ leads to the reduction of the *M* ^II^⋯Cd^II^ distance in **II** as compared to **I** [5.332 (1) and 4.945 (1) Å, respectively].

Similar deformed square-pyramidal coordination polyhedra (in some cases with disordering of the metal ion) have also been observed in several other five-coordinate complexes containing the [Zn(*L*)*X*] moiety (*X* = axial ligand) but were never found in complexes involving the [Ni(*L*)] fragment (see *Database survey*). The reasons for such differences have been considered in detail during analysis of the structure of the five-coordinate macrocyclic Zn^II^ complex with *X* = tetra­thio­anti­monato axial ligand and were explained mainly by preferable ligand field stabilization energy for the *d* ^8^ Ni^II^ electronic configuration as compared that for *d* ^10^ Zn^II^ (Näther *et al.*, 2022 ▸).

In general, the structure of the coordination polyhedron of the Zn^II^ ion in **II** has much in common with that discussed recently in detail for the [Zn(*L*)I]I_3_ complex (Gavrish *et al.*, 2021 ▸). In both compounds, the macrocyclic ligand *L* adopts the energetically favorable *trans*-III *R*,*R*,*S*,*S*) conformation (Bosnich *et al.*, 1965*a* ▸; Barefield *et al.*, 1986 ▸), though with some peculiarities connected with the displacement of the Zn^II^ ion from the mean N_4_ plane of the macrocycle donor atoms toward the coordinated iodide ion [0.336 (1) Å in **II** and 0.381 Å in triiodide complex]. In particular, the five-membered rings in **II** adopt *gauche–envelope* conformations with very similar bite angles [average value *ca* 83.5° (Table 1 ▸)]. The six-membered chelate rings in **II** are present in a chair conformation and differ from each other more significantly, both from the point of view of the Zn—N bond lengths and bite angles. So, the chelate ring in which the hydrogen atoms of the secondary amino groups have the same orientation as the displacement of the metal ion is characterized by smaller values of the Zn—N coordination bond lengths (average value 2.041 Å) and bite angle (*ca* 90°) as compared to the ring with the opposite orientation of the hydrogen atoms (average value 2.163 Å and *ca* 97°, respectively; Table 1 ▸). Similarly to [Zn(*L*)I]I_3_, a flattening of the former six-membered chelate ring at the Zn side is observed.

**Figure 2:**
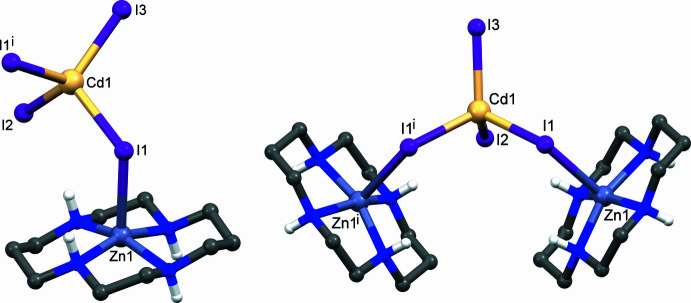
View of the two possible coordination modes of the [CdI_4_]^2−^ anion in **II**. Symmetry code: (i) *x*, −*y* + 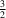 , *z*.

It should also be mentioned that the Zn—I1 distance to the symmetry-related I1(−*x* + 1, −*y* + 1, −*z* + 1) atom on the other side of the N_4_ plane is 3.579 (1) Å and this value seems to be too long for it to be considered as a coordination bond. This means that each component of the disordered Zn^II^ ion is truly five-coordinate. Therefore, the connectivity within the crystal is not uniquely defined and, in principle, the [CdI_4_]^2−^ anions can inter­act either with one or two [Zn(*L*)]^2+^ cations (Fig. 2 ▸).

## 3. Supra­molecular features

The N1—H⋯I2 inter­actions in both **I** and **II** together with either N1—H/N2—H⋯I1 hydrogen-bonding in **I** or Zn—I1 coordination in **II** determine close similarity in the mutual spatial arrangements of the cation and anion in both compounds (Fig. 1 ▸). As expected, the supra­molecular organization of the complexes under consideration is also very similar and is determined by the hydrogen-bonding inter­actions between the secondary amino groups of the ligand *L* in the [*M*(*L*)]^2+^ cations as the proton donors and I2 and I3 atoms of the [CdI_4_]^2−^ anions as the proton acceptors (Tables 2 ▸ and 3 ▸). Therefore, only complex **I** will be used for further illustration.

**Figure 3:**
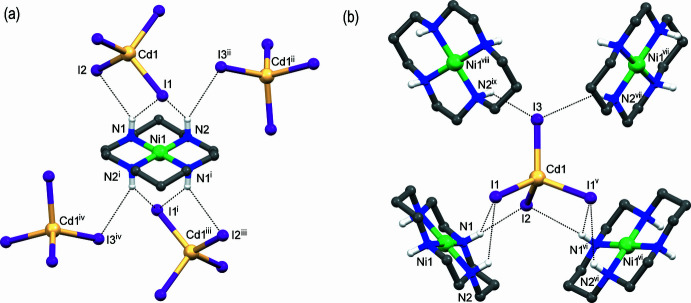
Nearest surrounding of the macrocyclic cation (*a*) and the anion (*b*) in **I** formed by N—H⋯I hydrogen bonding (black dashed lines). Symmetry codes: (i) −*x* + 1, −*y* + 1, −*z* + 1; (ii) *x* + 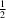 , −*y* + 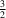 , −*z* + 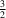 ; (iii) −*x* + 1, *y* − 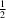 , −*z* + 1; (iv) −*x* + 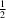 , −*y* + 1, *z* − 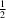 ; (v) *x*, −*y* + 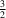 , *z*; (vi) −*x* + 1, *y* + 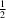 , −*z* + 1; (vii) *x* − 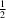 , −*y* + 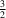 , −*z* + 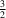 ; (viii) −*x* + 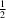 , −*y* + 1, *z* + 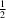 ; (ix) *x* − 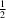 , *y*, −*z* + 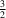 .

As a result of the hydrogen bonds N1—H⋯I2 and N2—H⋯I3, each macrocyclic cation [*M*(*L*)]^2+^ in **I** and **II** is surrounded by four [CdI_4_]^2−^ anions (Fig. 3 ▸ *a*). In turn, each of these iodide atoms forms two bonds with different macrocyclic cations, thus resulting in binding of four cations by a single anion (Fig. 3 ▸ *b*).

**Figure 4:**
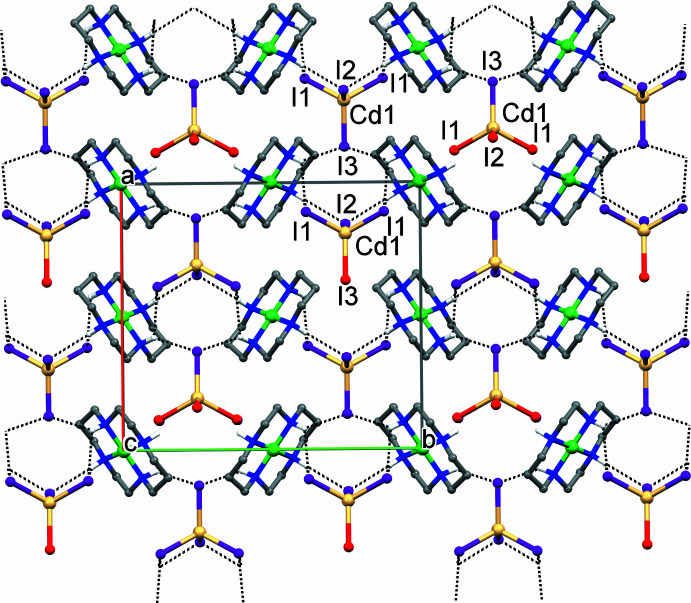
Fragment of the two-dimensional sheet in **I** parallel to the (101) plane as viewed along the *c* axis. Iodide atoms involved in the formation of sheets parallel to the ( 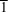 01) plane are shown in red. Hydrogen-bonding inter­actions are shown as dotted lines.

In the crystal, the alternating cations and anions form chains running along the *b*-axis direction that are arranged in two-dimensional sheets oriented parallel to the (101) and ( 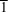 01) planes (Fig. 4 ▸). Since these sheets are built from the same cations and anions, this feature provides the three-dimensional coherence of crystals **I** and **II**.

## 4. Database survey

A search of the Cambridge Structural Database (CSD, version 5.44; Groom *et al.*, 2016 ▸) indicated that more than 20 compounds containing low-spin square-planar [Ni(*L*)]^2+^ cation have been characterized crystallographically. For all of them, relatively short Ni—N bond lengths in the equatorial planes typically not exceeding 1.97 Å and the absence of potential donor atoms in the axial positions of the Ni^II^ ion at distances shorter than 3.2 Å are inherent. Among them, several complexes containing a non-coordinated iodide anion as the counter-ion have also been described [CAFHUM (Prasad & McAuley, 1983 ▸); JIZTUH (Adam *et al.*, 1991 ▸); JIZTUH01–JIZTUH08 (Horii *et al.*, 2020 ▸)]. In general, the structural parameters of these compounds, in particular, the equatorial Ni—N bond lengths (1.93–1.96 Å) and Ni⋯I distances in the axial directions (3.29–3.34 Å) are very similar to those observed in **I**. Inter­estingly, there are two complexes formed by the [Ni(*L*)]^2+^ cation and tetra­hedral chloro­metalate anions [*M*Cl_4_]^2−^ with *M* = Zn^II^ (FAGWAL; Barefield *et al.*, 1986 ▸) and Ni^II^ (QASKOO; Heinemann *et al.*, 2022 ▸) that also demonstrate rather weak (if any) inter­action of the [Ni(*L*)]^2+^ cation with the halide [the Ni—Cl distances are 2.835 (average) and 3.305 Å, respectively].

In eight of the more than forty compounds containing the [Zn(*L*)]^2+^ cation that are present in the CSD, the Zn^II^ ion is five-coordinated in a square-pyramidal manner with different axial ligands including hexa­cyano­ferrate(III) (NEPYUC; Colacio *et al.*, 2001 ▸), thiol­ate (ICUFES and ICUFIW; Notni *et al.*, 2006 ▸), thio­anti­monate [GALPUI (Danker *et al.*, 2021 ▸) and KECVIB (Näther *et al.*, 2022 ▸)] as well as iodide [HEGNOW (Porai-Koshits *et al.*, 1994 ▸); JALBIL and JALBOR (Gavrish *et al.*, 2021 ▸)]. In all these five-coordinate complexes, the Zn^II^ atom is displaced from the mean N_4_ plane of the donor atoms of the macrocycle toward the axial ligand. Additionally, in some compounds (GALPUI, KECVIB and JALBOR), similar to **II**, some kind of disorder of the metal ion is also present. The Zn—I axial bond lengths of 2.66–2.77 Å observed in the iodide complexes are shorter than that found in **II** [2.8957 (11) Å].

A search of the CSD gives more than 90 hits related to the structural characterization of compounds containing the [CdI_4_]^2−^ anion. Like **I**, the majority of them are ionic species in which the charge of the anion is compensated by organic (*ca* 60 hits) or metalocomplex (*ca* 30 hits) cations. Besides, similarly to **II**, in three compounds that include the complex cations formed by Cd^II^ [ITAFAL (Satapathi *et al.*, 2011 ▸) and MATKUO (Seitz *et al.*, 2005 ▸)] or Cu^II^ (NEZXAS; Yu *et al.*, 2007 ▸), the tetra­iodo­cadmate anion displays the μ_2_-bridging function with the *M*—I coordination bonds shorter than 3.0 Å (*ca* 2.83, 2.97 and 2.76 Å, respectively). In general, regardless the nature of the cation and whether the [CdI_4_]^2−^ moiety is coordinated to the *M* ^II^ ion, it demonstrates a slightly distorted tetra­hedral shape similar to that observed in **I** and **II**.

## 5. Synthesis and crystallization

All chemicals and solvents used in this work were purchased from Sigma–Aldrich and were used without further purification. The complex [Ni(*L*)](ClO_4_)_2_ was prepared from ethanol solutions as described in the literature (Bosnich *et al.*, 1965*b* ▸). The complex [Zn(*L*)](ClO_4_)_2_ was prepared analogously by mixing of equimolar amounts of *L* and zinc perchlorate hexa­hydrate in ethanol.

[Ni(*L*)(CdI_4_)], **I**, was prepared as follows. [Ni(*L*)](ClO_4_)_2_ (50 mg, 0.11 mmol) was dissolved in 60 ml of an EtOH/H_2_O/DMF mixture (7:3:20 by volume). CdI_2_ (40 mg, 0.11 mmol) and KI (36 mg, 0.22 mmol) dissolved in 20 ml of an EtOH/H_2_O mixture (1:9 by volume) were added dropwise to this solution. Brown crystals formed in several days, were filtered off, washed with ethanol and dried in air. Yield: 22 mg (23%). Single crystals of **I** suitable for X-ray diffraction analysis were selected from the sample resulting from the synthesis.

Alternatively, complex **I** can be obtained using the chloride salt of Cd^II^. To 50 ml of an aqueous solution of CdCl_2_ (20 mg, 0.11 mmol) were added 0.4 ml of 57% aqueous HI and this mixture was added dropwise to a solution of [Ni(*L*)](ClO_4_)_2_ (50 mg, 0.11 mmol) in 40 ml of an EtOH/H_2_O mixture (3:1 by volume). Brown crystals formed in 5 days, were filtered off, washed with ethanol and dried in air. Yield: 35 mg (36%). Analysis calculated for C_10_H_24_CdI_4_N_4_Ni: C 13.66, H 2.75, N 6.37%. Found: C 13.78, H 2.60, N 6.42%.

[Zn(*L*)(CdI_4_)], **II**, was prepared similarly to **I**. [Zn(*L*)](ClO_4_)_2_ (52 mg, 0.11 mmol) was dissolved in 32 ml of an EtOH/H_2_O mixture (7:1 by volume). CdI_2_ (24 mg, 0.07 mmol) and KI (20 mg, 0.13 mmol) dissolved in 12 ml of an EtOH/H_2_O mixture (1:9 by volume) were added dropwise to this solution. Colorless crystals formed in several days, were filtered off, washed with ethanol and dried in air. Yield: 26 mg (46%). Analysis calculated for C_10_H_24_CdI_4_N_4_Zn: C 13.56, H 2.73, N 6.33%. Found: C 13.69, H 2.80, N 6.39%. Single crystals of **II** suitable for X-ray diffraction analysis were selected from the sample resulting from the synthesis.

## 6. Refinement

Crystal data, data collection and structure refinement details are summarized in Table 4 ▸. H atoms in **I** and **II** were placed in geometrically idealized positions and constrained to ride on their parent atoms, with C—H distances of methyl­ene H atoms of 0.97 Å (in **I**) or 0.99 Å (in **II**) and N—H distance of 0.91 Å with *U*_iso_(H) values of 1.2 *U*_eq_ of the parent atoms.

## Supplementary Material

Crystal structure: contains datablock(s) I, II. DOI: 10.1107/S2056989023007004/ex2074sup1.cif

Structure factors: contains datablock(s) I. DOI: 10.1107/S2056989023007004/ex2074Isup2.hkl

Structure factors: contains datablock(s) II. DOI: 10.1107/S2056989023007004/ex2074IIsup3.hkl

CCDC references: 2281090, 2281091

Additional supporting information:  crystallographic information; 3D view; checkCIF report

## Figures and Tables

**Table 1:** Selected geometric parameters (Å, °)

**I**		**II**	
Ni1—N1	1.940 (4)	Zn1—N1	2.157 (4)
Ni1—N2	1.943 (4)	Zn1—N2	2.169 (4)
		Zn1—N1^i^	2.027 (4)
		Zn1—N2^i^	2.053 (4)
		Zn1—I1	2.8957 (11)
Cd1—I1	2.7825 (4)	Cd1—I1	2.8208 (5)
Cd1—I2	2.8024 (7)	Cd1—I2	2.7756 (8)
Cd1—I3	2.7615 (7)	Cd1—I3	2.7442 (7)
			
N1—Ni1—N2^i^	86.35 (16)	N1—Zn1—N2^i^	83.73 (17)
		N1i—Zn1—N2	83.43 (17)
N1—Ni1—N2	93.65 (16)	N1—Zn1—N2	97.02 (18)
		N1i—Zn1—N2i	89.93 (16)
I1—Cd1—I1^ii^	108.39 (2)	I1—Cd1—I1^ii^	106.04 (2)
I1—Cd1—I2	106.608 (15)	I1—Cd1—I2	107.978 (16)
I1—Cd1—I3	111.407 (15)	I1—Cd1—I3	110.135 (17)
I2—Cd1—I3	112.16 (2)	I2—Cd1—I3	114.22 (3)

Symmetry codes: (i) −*x* + 1, −*y* + 1, −*z* + 1; (ii) *x*, −*y* + 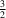 , *z*.

**Table 2:** Hydrogen-bond geometry (Å, °) for **I** [Chem scheme1]

*D*—H⋯*A*	*D*—H	H⋯*A*	*D*⋯*A*	*D*—H⋯*A*
N1—H1⋯I1	0.91	3.22	3.829 (4)	127
N2—H2⋯I1	0.91	3.15	3.768 (4)	126
N1—H1⋯I2	0.91	3.03	3.742 (4)	137
N2—H2⋯I3^i^	0.91	3.14	3.881 (4)	140

Symmetry code: (i) 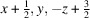 .

**Table 3:** Hydrogen-bond geometry (Å, °) for **II** [Chem scheme1]

*D*—H⋯*A*	*D*—H	H⋯*A*	*D*⋯*A*	*D*—H⋯*A*
N1—H1⋯I2	0.91	2.95	3.714 (4)	142
N2—H2⋯I3^i^	0.91	3.11	3.871 (4)	143

Symmetry code: (i) 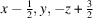 .

**Table 4:** Experimental details

	**I**	**II**
Crystal data		
Chemical formula	[Ni(C_10_H_24_N_4_)][CdI_4_]	[CdZnI_4_(C_10_H_24_N_4_)]
*M*_r_	879.04	885.70
Crystal system, space group	Orthorhombic, *P* *n* *m* *a*	Orthorhombic, *P* *n* *m* *a*
Temperature (K)	200	293
*a*, *b*, *c* (Å)	15.4317 (3), 17.2945 (3), 7.98733 (15)	15.6013 (3), 17.2644 (3), 8.1099 (2)
*V* (Å^3^)	2131.69 (7)	2184.38 (8)
*Z*	4	4
Radiation type	Mo *K*α	Mo *K*α
μ (mm^−1^)	7.67	7.72
Crystal size (mm)	0.1 × 0.05 × 0.03	0.15 × 0.1 × 0.1

Data collection		
Diffractometer	Rigaku Xcalibur Eos	Rigaku Xcalibur Eos
Absorption correction	Multi-scan (*CrysAlis PRO*; Rigaku OD, 2022 ▸)	Multi-scan (*CrysAlis PRO*; Rigaku OD, 2022 ▸)
*T*_min_, *T*_max_	0.573, 1.000	0.426, 1.000
No. of measured, independent and observed [*I* > 2σ(*I*)] reflections	16993, 2644, 2204	9158, 2582, 2096
*R*_int_	0.044	0.031
(sin θ/λ)_max_ (Å^−1^)	0.667	0.666

Refinement		
*R*[*F* ^2^ > 2σ(*F* ^2^)], *wR*(*F* ^2^), *S*	0.031, 0.064, 1.06	0.032, 0.065, 1.04
No. of reflections	2644	2582
No. of parameters	97	100
H-atom treatment	H-atom parameters constrained	H-atom parameters constrained
Δρ_max_, Δρ_min_ (e Å^−3^)	2.57, −1.23	1.36, −0.94

Computer programs: *CrysAlis PRO* (Rigaku OD, 2022 ▸), *SHELXT* (Sheldrick, 2015*a* ▸), *SHELXL2018/3* (Sheldrick, 2015*b* ▸), *Mercury* (Macrae *et al.*, 2020 ▸) and *publCIF* (Westrip, 2010 ▸).

## supplementary crystallographic information

### (1,4,8,11-Tetraazacyclotetradecane-κ4N)nickel(II) tetraiodidocadmate(II) (I) Crystal data

**Table:**

[Ni(C_10_H_24_N_4_)][CdI_4_]	*D*_x_ = 2.739 Mg m^−^^3^
*M**_r_* = 879.04	Mo *K*α radiation, λ = 0.71073 Å
Orthorhombic, *P**n**m**a*	Cell parameters from 5956 reflections
*a* = 15.4317 (3) Å	θ = 2.4–28.8°
*b* = 17.2945 (3) Å	µ = 7.67 mm^−^^1^
*c* = 7.98733 (15) Å	*T* = 200 K
*V* = 2131.69 (7) Å^3^	Prism, clear dark orange
*Z* = 4	0.1 × 0.05 × 0.03 mm
*F*(000) = 1600	

### (1,4,8,11-Tetraazacyclotetradecane-κ4N)nickel(II) tetraiodidocadmate(II) (I) Data collection

**Table:**

Rigaku Xcalibur Eos diffractometer	2644 independent reflections
Radiation source: fine-focus sealed X-ray tube, Enhance (Mo) X-ray Source	2204 reflections with *I* > 2σ(*I*)
Graphite monochromator	*R*_int_ = 0.044
Detector resolution: 16.1593 pixels mm^-1^	θ_max_ = 28.3°, θ_min_ = 2.4°
ω scans	*h* = −19→19
Absorption correction: multi-scan (CrysAlisPro; Rigaku OD, 2022)	*k* = −23→23
*T*_min_ = 0.573, *T*_max_ = 1.000	*l* = −10→10
16993 measured reflections	

### (1,4,8,11-Tetraazacyclotetradecane-κ4N)nickel(II) tetraiodidocadmate(II) (I) Refinement

**Table:**

Refinement on *F*^2^	Primary atom site location: dual
Least-squares matrix: full	Hydrogen site location: mixed
*R*[*F*^2^ > 2σ(*F*^2^)] = 0.031	H-atom parameters constrained
*wR*(*F*^2^) = 0.064	*w* = 1/[σ^2^(*F*_o_^2^) + (0.0237*P*)^2^ + 2.5375*P*] where *P* = (*F*_o_^2^ + 2*F*_c_^2^)/3
*S* = 1.06	(Δ/σ)_max_ = 0.001
2644 reflections	Δρ_max_ = 2.57 e Å^−^^3^
97 parameters	Δρ_min_ = −1.23 e Å^−^^3^
0 restraints	

### (1,4,8,11-Tetraazacyclotetradecane-κ4N)nickel(II) tetraiodidocadmate(II) (I) Special details

**Table:**

Geometry. All esds (except the esd in the dihedral angle between two l.s. planes) are estimated using the full covariance matrix. The cell esds are taken into account individually in the estimation of esds in distances, angles and torsion angles; correlations between esds in cell parameters are only used when they are defined by crystal symmetry. An approximate (isotropic) treatment of cell esds is used for estimating esds involving l.s. planes.

### (1,4,8,11-Tetraazacyclotetradecane-κ4N)nickel(II) tetraiodidocadmate(II) (I) Fractional atomic coordinates and isotropic or equivalent isotropic displacement parameters (Å^2^)

**Table:**

	*x*	*y*	*z*	*U*_iso_*/*U*_eq_	
I1	0.38762 (2)	0.61952 (2)	0.75107 (4)	0.02581 (10)	
I3	0.13165 (3)	0.750000	0.68459 (7)	0.03244 (13)	
I2	0.34261 (3)	0.750000	0.27469 (6)	0.02831 (12)	
Cd1	0.30734 (3)	0.750000	0.61887 (7)	0.02466 (13)	
Ni1	0.500000	0.500000	0.500000	0.01715 (18)	
N2	0.6010 (2)	0.5611 (2)	0.5599 (5)	0.0224 (8)	
H2	0.577167	0.604133	0.607128	0.027*	
N1	0.4693 (2)	0.5664 (2)	0.3133 (5)	0.0219 (8)	
H1	0.448687	0.609197	0.366795	0.026*	
C2	0.6554 (3)	0.5152 (3)	0.6750 (7)	0.0325 (12)	
H2A	0.694792	0.549462	0.738857	0.039*	
H2B	0.690905	0.477600	0.611403	0.039*	
C1	0.4050 (3)	0.5265 (3)	0.2080 (7)	0.0335 (12)	
H1A	0.434068	0.489215	0.132448	0.040*	
H1B	0.372638	0.564247	0.139112	0.040*	
C5	0.6550 (3)	0.5965 (3)	0.4269 (7)	0.0316 (12)	
H5A	0.682907	0.555079	0.360435	0.038*	
H5B	0.701410	0.627786	0.479260	0.038*	
C4	0.6021 (4)	0.6473 (3)	0.3119 (7)	0.0366 (13)	
H4A	0.641638	0.675793	0.236207	0.044*	
H4B	0.570150	0.685815	0.379726	0.044*	
C3	0.5377 (3)	0.6012 (3)	0.2072 (6)	0.0294 (11)	
H3A	0.510613	0.635643	0.123246	0.035*	
H3B	0.568819	0.559713	0.146499	0.035*	

### (1,4,8,11-Tetraazacyclotetradecane-κ4N)nickel(II) tetraiodidocadmate(II) (I) Atomic displacement parameters (Å^2^)

**Table:**

	*U*^11^	*U*^22^	*U*^33^	*U*^12^	*U*^13^	*U*^23^
I1	0.02646 (18)	0.02180 (17)	0.0292 (2)	0.00438 (12)	0.00288 (13)	0.00314 (13)
I3	0.0221 (2)	0.0352 (3)	0.0400 (3)	0.000	0.0040 (2)	0.000
I2	0.0290 (3)	0.0274 (2)	0.0285 (3)	0.000	−0.0001 (2)	0.000
Cd1	0.0227 (3)	0.0198 (2)	0.0315 (3)	0.000	0.0013 (2)	0.000
Ni1	0.0171 (4)	0.0164 (4)	0.0179 (4)	−0.0020 (3)	−0.0014 (3)	0.0013 (3)
N2	0.018 (2)	0.021 (2)	0.029 (2)	−0.0005 (15)	0.0024 (17)	−0.0012 (17)
N1	0.026 (2)	0.0195 (19)	0.020 (2)	0.0035 (16)	−0.0001 (17)	−0.0008 (16)
C2	0.025 (3)	0.040 (3)	0.033 (3)	−0.003 (2)	−0.010 (2)	−0.003 (2)
C1	0.032 (3)	0.039 (3)	0.029 (3)	0.003 (2)	−0.010 (2)	0.003 (2)
C5	0.023 (3)	0.029 (3)	0.042 (3)	−0.011 (2)	0.001 (2)	0.000 (2)
C4	0.043 (3)	0.022 (3)	0.045 (3)	−0.006 (2)	0.012 (3)	0.004 (2)
C3	0.032 (3)	0.026 (3)	0.030 (3)	−0.001 (2)	0.008 (2)	0.007 (2)

### (1,4,8,11-Tetraazacyclotetradecane-κ4N)nickel(II) tetraiodidocadmate(II) (I) Geometric parameters (Å, º)

**Table:**

I1—Cd1	2.7825 (4)	C2—H2A	0.9900
I1—Ni1	3.3618 (3)	C2—H2B	0.9900
I3—Cd1	2.7615 (7)	C2—C1^i^	1.504 (7)
I2—Cd1	2.8024 (7)	C1—H1A	0.9900
Ni1—N2	1.943 (4)	C1—H1B	0.9900
Ni1—N2^i^	1.943 (4)	C5—H5A	0.9900
Ni1—N1^i^	1.940 (4)	C5—H5B	0.9900
Ni1—N1	1.940 (4)	C5—C4	1.511 (7)
N2—H2	0.9124	C4—H4A	0.9900
N2—C2	1.477 (6)	C4—H4B	0.9900
N2—C5	1.483 (6)	C4—C3	1.524 (7)
N1—H1	0.9125	C3—H3A	0.9900
N1—C1	1.472 (6)	C3—H3B	0.9900
N1—C3	1.481 (6)		
			
Cd1—I1—Ni1	120.129 (15)	N2—C2—H2B	110.3
I1^ii^—Cd1—I1	108.39 (2)	N2—C2—C1^i^	106.9 (4)
I1^ii^—Cd1—I2	106.608 (15)	H2A—C2—H2B	108.6
I1—Cd1—I2	106.608 (15)	C1^i^—C2—H2A	110.3
I3—Cd1—I1	111.407 (15)	C1^i^—C2—H2B	110.3
I3—Cd1—I1^ii^	111.407 (15)	N1—C1—C2^i^	106.7 (4)
I3—Cd1—I2	112.16 (2)	N1—C1—H1A	110.4
N2—Ni1—I1	86.14 (11)	N1—C1—H1B	110.4
N2^i^—Ni1—I1	93.86 (11)	C2^i^—C1—H1A	110.4
N2^i^—Ni1—N2	180.0	C2^i^—C1—H1B	110.4
N1^i^—Ni1—I1	91.78 (11)	H1A—C1—H1B	108.6
N1—Ni1—I1	88.22 (11)	N2—C5—H5A	109.2
N1—Ni1—N2^i^	86.35 (16)	N2—C5—H5B	109.2
N1^i^—Ni1—N2^i^	93.65 (16)	N2—C5—C4	111.9 (4)
N1^i^—Ni1—N2	86.35 (16)	H5A—C5—H5B	107.9
N1—Ni1—N2	93.65 (16)	C4—C5—H5A	109.2
N1^i^—Ni1—N1	180.0	C4—C5—H5B	109.2
Ni1—N2—H2	102.8	C5—C4—H4A	109.1
C2—N2—Ni1	108.5 (3)	C5—C4—H4B	109.1
C2—N2—H2	114.3	C5—C4—C3	112.4 (4)
C2—N2—C5	110.4 (4)	H4A—C4—H4B	107.9
C5—N2—Ni1	119.9 (3)	C3—C4—H4A	109.1
C5—N2—H2	100.7	C3—C4—H4B	109.1
Ni1—N1—H1	101.9	N1—C3—C4	111.3 (4)
C1—N1—Ni1	109.0 (3)	N1—C3—H3A	109.4
C1—N1—H1	114.4	N1—C3—H3B	109.4
C1—N1—C3	110.1 (4)	C4—C3—H3A	109.4
C3—N1—Ni1	120.4 (3)	C4—C3—H3B	109.4
C3—N1—H1	100.7	H3A—C3—H3B	108.0
N2—C2—H2A	110.3		
			
Ni1—N2—C2—C1^i^	−39.7 (5)	C2—N2—C5—C4	−177.0 (4)
Ni1—N2—C5—C4	55.7 (5)	C1—N1—C3—C4	176.4 (4)
Ni1—N1—C1—C2^i^	39.1 (5)	C5—N2—C2—C1^i^	−173.1 (4)
Ni1—N1—C3—C4	−55.5 (5)	C5—C4—C3—N1	66.9 (5)
N2—C5—C4—C3	−67.2 (6)	C3—N1—C1—C2^i^	173.2 (4)

Symmetry codes: (i) −*x*+1, −*y*+1, −*z*+1; (ii) *x*, −*y*+3/2, *z*.

### (1,4,8,11-Tetraazacyclotetradecane-κ4N)nickel(II) tetraiodidocadmate(II) (I) Hydrogen-bond geometry (Å, º)

**Table:**

*D*—H···*A*	*D*—H	H···*A*	*D*···*A*	*D*—H···*A*
N1—H1···I1	0.91	3.22	3.829 (4)	127
N2—H2···I1	0.91	3.15	3.768 (4)	126
N1—H1···I2	0.91	3.03	3.742 (4)	137
N2—H2···I3^iii^	0.91	3.14	3.881 (4)	140

Symmetry code: (iii) *x*+1/2, *y*, −*z*+3/2.

### Triiodido-1κ3I-µ-iodido-(1,4,8,11-tetraazacyclotetradecane-2κ4N)cadmium(II)zinc(II) (II) Crystal data

**Table:**

[CdZnI_4_(C_10_H_24_N_4_)]	*D*_x_ = 2.693 Mg m^−^^3^
*M**_r_* = 885.70	Mo *K*α radiation, λ = 0.71073 Å
Orthorhombic, *P**n**m**a*	Cell parameters from 3723 reflections
*a* = 15.6013 (3) Å	θ = 2.4–28.5°
*b* = 17.2644 (3) Å	µ = 7.72 mm^−^^1^
*c* = 8.1099 (2) Å	*T* = 293 K
*V* = 2184.38 (8) Å^3^	Prism, clear light colourless
*Z* = 4	0.15 × 0.1 × 0.1 mm
*F*(000) = 1608	

### Triiodido-1κ3I-µ-iodido-(1,4,8,11-tetraazacyclotetradecane-2κ4N)cadmium(II)zinc(II) (II) Data collection

**Table:**

Rigaku Xcalibur Eos diffractometer	2582 independent reflections
Radiation source: fine-focus sealed X-ray tube, Enhance (Mo) X-ray Source	2096 reflections with *I* > 2σ(*I*)
Graphite monochromator	*R*_int_ = 0.031
Detector resolution: 16.1593 pixels mm^-1^	θ_max_ = 28.3°, θ_min_ = 2.4°
ω scans	*h* = −20→18
Absorption correction: multi-scan (CrysAlisPro; Rigaku OD, 2022)	*k* = −20→21
*T*_min_ = 0.426, *T*_max_ = 1.000	*l* = −10→9
9158 measured reflections	

### Triiodido-1κ3I-µ-iodido-(1,4,8,11-tetraazacyclotetradecane-2κ4N)cadmium(II)zinc(II) (II) Refinement

**Table:**

Refinement on *F*^2^	Primary atom site location: dual
Least-squares matrix: full	Hydrogen site location: mixed
*R*[*F*^2^ > 2σ(*F*^2^)] = 0.032	H-atom parameters constrained
*wR*(*F*^2^) = 0.065	*w* = 1/[σ^2^(*F*_o_^2^) + (0.0226*P*)^2^ + 2.0518*P*] where *P* = (*F*_o_^2^ + 2*F*_c_^2^)/3
*S* = 1.04	(Δ/σ)_max_ = 0.001
2582 reflections	Δρ_max_ = 1.36 e Å^−^^3^
100 parameters	Δρ_min_ = −0.94 e Å^−^^3^
0 restraints	

### Triiodido-1κ3I-µ-iodido-(1,4,8,11-tetraazacyclotetradecane-2κ4N)cadmium(II)zinc(II) (II) Special details

**Table:**

Geometry. All esds (except the esd in the dihedral angle between two l.s. planes) are estimated using the full covariance matrix. The cell esds are taken into account individually in the estimation of esds in distances, angles and torsion angles; correlations between esds in cell parameters are only used when they are defined by crystal symmetry. An approximate (isotropic) treatment of cell esds is used for estimating esds involving l.s. planes.

### Triiodido-1κ3I-µ-iodido-(1,4,8,11-tetraazacyclotetradecane-2κ4N)cadmium(II)zinc(II) (II) Fractional atomic coordinates and isotropic or equivalent isotropic displacement parameters (Å^2^)

**Table:**

	*x*	*y*	*z*	*U*_iso_*/*U*_eq_	Occ. (<1)
I1	0.60630 (2)	0.61948 (2)	0.72972 (4)	0.04273 (11)	
I2	0.65629 (3)	0.750000	0.25522 (7)	0.04439 (14)	
I3	0.85889 (3)	0.750000	0.67325 (8)	0.04858 (15)	
Cd1	0.68815 (3)	0.750000	0.59194 (7)	0.04059 (15)	
Zn1	0.50999 (12)	0.51552 (8)	0.5195 (2)	0.0325 (4)	0.5
N1	0.5302 (3)	0.5688 (2)	0.3001 (5)	0.0365 (10)	
H1	0.553580	0.612411	0.344223	0.044*	
N2	0.3925 (2)	0.5640 (2)	0.5681 (5)	0.0382 (10)	
H2	0.413021	0.607917	0.616123	0.046*	
C1	0.5928 (4)	0.5255 (3)	0.2012 (6)	0.0472 (14)	
H1A	0.623595	0.560764	0.129340	0.057*	
H1B	0.563348	0.487810	0.132915	0.057*	
C2	0.3449 (3)	0.5153 (3)	0.6856 (8)	0.0504 (15)	
H2A	0.310992	0.477403	0.626059	0.060*	
H2B	0.306174	0.547248	0.750024	0.060*	
C3	0.4573 (3)	0.5993 (3)	0.2050 (6)	0.0437 (13)	
H3A	0.427594	0.556651	0.152125	0.052*	
H3B	0.478559	0.633453	0.119263	0.052*	
C4	0.3943 (4)	0.6438 (3)	0.3148 (8)	0.0552 (16)	
H4A	0.426472	0.681033	0.379528	0.066*	
H4B	0.355727	0.672860	0.244098	0.066*	
C5	0.3405 (3)	0.5950 (3)	0.4320 (7)	0.0477 (14)	
H5A	0.294530	0.626459	0.476734	0.057*	
H5B	0.314889	0.552420	0.371555	0.057*	

### Triiodido-1κ3I-µ-iodido-(1,4,8,11-tetraazacyclotetradecane-2κ4N)cadmium(II)zinc(II) (II) Atomic displacement parameters (Å^2^)

**Table:**

	*U*^11^	*U*^22^	*U*^33^	*U*^12^	*U*^13^	*U*^23^
I1	0.0457 (2)	0.03772 (19)	0.0448 (2)	−0.01092 (14)	−0.00578 (17)	0.00554 (15)
I2	0.0440 (3)	0.0406 (3)	0.0485 (3)	0.000	0.0018 (2)	0.000
I3	0.0348 (3)	0.0515 (3)	0.0595 (4)	0.000	−0.0069 (3)	0.000
Cd1	0.0354 (3)	0.0320 (3)	0.0543 (4)	0.000	−0.0033 (3)	0.000
Zn1	0.0297 (9)	0.0377 (11)	0.0302 (9)	0.0088 (7)	0.0030 (7)	0.0069 (7)
N1	0.043 (2)	0.034 (2)	0.032 (2)	−0.0040 (18)	−0.004 (2)	0.0008 (17)
N2	0.032 (2)	0.036 (2)	0.046 (3)	0.0004 (17)	−0.002 (2)	−0.0030 (19)
C1	0.054 (3)	0.053 (3)	0.035 (3)	−0.011 (3)	0.017 (3)	−0.002 (2)
C2	0.035 (3)	0.050 (3)	0.066 (4)	0.000 (2)	0.016 (3)	−0.005 (3)
C3	0.053 (3)	0.040 (3)	0.038 (3)	−0.003 (2)	−0.010 (3)	0.010 (2)
C4	0.062 (4)	0.038 (3)	0.066 (4)	0.011 (3)	−0.027 (3)	0.007 (3)
C5	0.034 (3)	0.045 (3)	0.064 (4)	0.009 (2)	−0.005 (3)	−0.003 (3)

### Triiodido-1κ3I-µ-iodido-(1,4,8,11-tetraazacyclotetradecane-2κ4N)cadmium(II)zinc(II) (II) Geometric parameters (Å, º)

**Table:**

I1—Cd1	2.8208 (5)	C1—H1A	0.9700
I1—Zn1	2.8957 (11)	C1—H1B	0.9700
I2—Cd1	2.7756 (8)	C1—C2^i^	1.512 (8)
I3—Cd1	2.7442 (7)	C2—H2A	0.9700
Zn1—N1	2.027 (4)	C2—H2B	0.9700
Zn1—N1^i^	2.157 (4)	C3—H3A	0.9700
Zn1—N2^i^	2.169 (4)	C3—H3B	0.9700
Zn1—N2	2.053 (4)	C3—C4	1.534 (8)
N1—H1	0.9100	C4—H4A	0.9700
N1—C1	1.468 (6)	C4—H4B	0.9700
N1—C3	1.471 (6)	C4—C5	1.522 (8)
N2—H2	0.9101	C5—H5A	0.9700
N2—C2	1.472 (6)	C5—H5B	0.9700
N2—C5	1.470 (6)		
			
Cd1—I1—Zn1	119.79 (4)	C5—N2—Zn1^i^	111.8 (3)
I1—Cd1—I1^ii^	106.04 (2)	C5—N2—H2	102.3
I2—Cd1—I1	107.978 (16)	C5—N2—C2	114.5 (4)
I2—Cd1—I1^ii^	107.978 (16)	N1—C1—H1A	109.8
I3—Cd1—I1^ii^	110.135 (17)	N1—C1—H1B	109.8
I3—Cd1—I1	110.135 (17)	N1—C1—C2^i^	109.5 (4)
I3—Cd1—I2	114.22 (3)	H1A—C1—H1B	108.2
N1^i^—Zn1—I1	99.78 (12)	C2^i^—C1—H1A	109.8
N1—Zn1—I1	98.91 (12)	C2^i^—C1—H1B	109.8
N1—Zn1—N1^i^	161.17 (7)	N2—C2—C1^i^	109.5 (4)
N1—Zn1—N2^i^	83.73 (17)	N2—C2—H2A	109.8
N1^i^—Zn1—N2^i^	89.93 (16)	N2—C2—H2B	109.8
N1—Zn1—N2	97.02 (18)	C1^i^—C2—H2A	109.8
N2—Zn1—I1	95.59 (12)	C1^i^—C2—H2B	109.8
N2^i^—Zn1—I1	102.79 (12)	H2A—C2—H2B	108.2
N2—Zn1—N1^i^	83.43 (17)	N1—C3—H3A	109.3
N2—Zn1—N2^i^	161.28 (6)	N1—C3—H3B	109.3
Zn1—N1—Zn1^i^	18.83 (7)	N1—C3—C4	111.8 (4)
Zn1^i^—N1—H1	114.1	H3A—C3—H3B	107.9
Zn1—N1—H1	95.3	C4—C3—H3A	109.3
C1—N1—Zn1^i^	102.7 (3)	C4—C3—H3B	109.3
C1—N1—Zn1	110.6 (3)	C3—C4—H4A	108.3
C1—N1—H1	111.7	C3—C4—H4B	108.3
C1—N1—C3	114.2 (4)	H4A—C4—H4B	107.4
C3—N1—Zn1^i^	111.9 (3)	C5—C4—C3	116.0 (4)
C3—N1—Zn1	120.2 (3)	C5—C4—H4A	108.3
C3—N1—H1	102.7	C5—C4—H4B	108.3
Zn1—N2—Zn1^i^	18.72 (6)	N2—C5—C4	111.5 (4)
Zn1^i^—N2—H2	114.9	N2—C5—H5A	109.3
Zn1—N2—H2	96.2	N2—C5—H5B	109.3
C2—N2—Zn1^i^	101.8 (3)	C4—C5—H5A	109.3
C2—N2—Zn1	110.0 (3)	C4—C5—H5B	109.3
C2—N2—H2	112.1	H5A—C5—H5B	108.0
C5—N2—Zn1	119.8 (3)		
			
Zn1—N1—C1—C2^i^	−30.3 (5)	Zn1—N2—C5—C4	−45.3 (5)
Zn1^i^—N1—C1—C2^i^	−48.2 (4)	N1—C3—C4—C5	−71.7 (6)
Zn1^i^—N1—C3—C4	63.8 (5)	C1—N1—C3—C4	180.0 (4)
Zn1—N1—C3—C4	45.0 (5)	C2—N2—C5—C4	−179.3 (4)
Zn1^i^—N2—C2—C1^i^	48.1 (5)	C3—N1—C1—C2^i^	−169.5 (4)
Zn1—N2—C2—C1^i^	30.6 (5)	C3—C4—C5—N2	71.8 (6)
Zn1^i^—N2—C5—C4	−64.2 (5)	C5—N2—C2—C1^i^	168.9 (4)

Symmetry codes: (i) −*x*+1, −*y*+1, −*z*+1; (ii) *x*, −*y*+3/2, *z*.

### Triiodido-1κ3I-µ-iodido-(1,4,8,11-tetraazacyclotetradecane-2κ4N)cadmium(II)zinc(II) (II) Hydrogen-bond geometry (Å, º)

**Table:**

*D*—H···*A*	*D*—H	H···*A*	*D*···*A*	*D*—H···*A*
N1—H1···I2	0.91	2.95	3.714 (4)	142
N2—H2···I3^iii^	0.91	3.11	3.871 (4)	143

Symmetry code: (iii) *x*−1/2, *y*, −*z*+3/2.
